# Bone Structure and Turnover in Postmenopausal Women With Long‐Standing Type 1 Diabetes

**DOI:** 10.1002/jbm4.10831

**Published:** 2023-10-15

**Authors:** Viral N Shah, Shijing Qui, Jason Stoneback, Lubna Qamar, Virginia L Ferguson, Wendy M Kohrt, Janet K Snell‐Bergeon, Sudhaker D Rao

**Affiliations:** ^1^ Barbara Davis Center for Diabetes University of Colorado Anschutz Medical Campus Aurora CO USA; ^2^ Division of Endocrinology, Diabetes, and Bone & Mineral Disorders, Bone and Mineral Research Laboratory Henry Ford Health Detroit MI USA; ^3^ Department of Orthopedics University of Colorado Anschutz Medical Campus Aurora CO USA; ^4^ Department of Mechanical Engineering University of Colorado Boulder CO USA; ^5^ Department of Geriatrics University of Colorado Anschutz Medical Campus Aurora CO USA

**Keywords:** BONE FORMATION, BONE HISTOMORPHOMETRY, BONE STRUCTURE, BONE TURNOVER, TYPE 1 DIABETES

## Abstract

Compromised bone structural and mechanical properties are implicated in the increased fracture risk in type 1 diabetes (T1D). We investigated bone structure and turnover by histomorphometry in postmenopausal women with T1D and controls without diabetes using tetracycline double‐labeled transiliac bone biopsy. After in vivo tetracycline double labeling, postmenopausal women with T1D of at least 10 years and without diabetes underwent transiliac bone biopsy. An expert blinded to the study group performed histomorphometry. Static and dynamic histomorphometry measurements were performed and compared between the two groups. The analysis included 9 postmenopausal women with T1D (mean age 58.4 ± 7.1 years with 37.9 ± 10.9 years of diabetes and HbA1c 7.1% ± 0.4%) and 7 postmenopausal women without diabetes (mean age 60.9 ± 3.3 years and HbA1c 5.4% ± 0.2%). There were no significant differences in serum PTH (38.6 ± 8.1 versus 51.9 ± 23.9 pg/mL), CTX (0.4 ± 0.2 versus 0.51 ± 0.34 ng/mL), or P1NP (64.5 ± 26.2 versus 87.3 ± 45.3 ng/mL). Serum 25‐hydroxyvitamin D levels were higher in T1D than in controls (53.1 ± 20.8 versus 30.9 ± 8.2 ng/mL, *p* < 0.05). Bone structure metrics (bone volume, trabecular thickness, trabecular number, and cortical thickness) were similar between the groups. Indices of bone formation (osteoid volume, osteoid surface, and bone formation rate) were 40% lower in T1D and associated with lower activation frequency. However, the differences in bone formation were not statistically significant. Long‐standing T1D may affect bone turnover, mainly bone formation, without significantly affecting bone structure. Further research is needed to understand bone turnover and factors affecting bone turnover in people with T1D. © 2023 The Authors. *JBMR Plus* published by Wiley Periodicals LLC. on behalf of American Society for Bone and Mineral Research.

## Graphical abstract

Bone Turnover in Postmenopausal women with T1D.

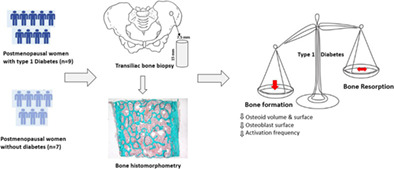

## Introduction

Fracture risk is four‐ to sixfold higher in people with type 1 diabetes (T1D).^(^ ^1^, ^2^ ^)^ Studies have reported normal or modestly reduced bone mineral density (BMD) by dual‐energy X‐ray absorptiometry (DXA) in people with T1D compared with controls without diabetes.^(^ ^3^, ^4^ ^)^ Therefore, the increased magnitude of fracture risk in people with T1D is believed to be related to compromised bone structural and material properties.

Diabetes (both T1D and type 2 diabetes [T2D]) is characterized by low bone turnover.^(^ ^5^ ^)^ Chronic hyperglycemia and peripheral hyperinsulinemia (mainly in T2D) may lead to suppression of bone formation and resorption.^(^ ^6^, ^7^, ^8^ ^)^ Animal studies using streptozotocin (STZ)‐induced insulin deficiency mouse and rat models have reported low bone formation, reduced trabecular bone and osteoid, and decreased mineral apposition rate, where most of these deficits were corrected by insulin administration, suggesting a role of hyperglycemia in suppression of bone turnover.^(^ ^9^, ^10^, ^11^, ^12^ ^)^ However, data on bone turnover using the gold standard bone histomorphometry technique in people with T1D are scant.

A study by Krakauer and colleagues reported decreased bone formation rate in the cortical and trabecular bone in people with diabetes (6 subjects with T2D and 2 subjects with T1D) compared with premenopausal females without diabetes.^(^ ^13^ ^)^ Clinical interpretation of this study is limited because of inclusion of only two subjects with T1D. Another study by Armas and colleagues in 18 patients with T1D, compared with 18 matched controls without diabetes, failed to show any differences in histomorphometric or micro‐CT measurements.^(^ ^14^ ^)^ Participants with T1D in the study were younger (mean age 31 years) with shorter duration of disease (mean diabetes duration 15 years) and well‐controlled diabetes (mean A1c of 6.8%).^(^ ^14^ ^)^

Considering the conflicting results of previous studies and their limitations, we aimed to evaluate bone structure and turnover by bone histomorphometry in postmenopausal women with long‐standing T1D.

## Materials and Methods

### Study design

This is a cross‐sectional, exploratory analysis from the parent study aimed to evaluate bone structural (*n* = 30 per group) and tissue material properties (*n* = 10 per group) in postmenopausal women with T1D and postmenopausal women without diabetes. The present analysis only focuses on bone histomorphometry analysis by diabetes status. Colorado Multiple Institutional Review Board approved the study.

### Patient selection

Postmenopausal women with T1D with diabetes duration of at least 10 years and postmenopausal women without diabetes were invited to participate in this study. T1D was defined as currently on insulin therapy, diagnosed before age 30 years, and immediately on insulin therapy or a clinical course consistent with T1D.^(^ ^15^ ^)^ Controls were defined as no medical history of diabetes with HbA1c <5.7% as published previously.^(^ ^16^, ^17^ ^)^ Menopause was defined as no menstrual periods for at least 12 consecutive months, a history of hysterectomy with bilateral oophorectomy, or a history of hysterectomy without oophorectomy and with serum follicle‐stimulating hormone (FSH) >40 IU/L.

Patients with history of malabsorption syndrome, rheumatologic disease, parathyroid disease, cancer other than skin cancer, oral or injectable steroid intake for >3 months, use of an immunosuppressant in the past 2 years, chronic kidney disease (estimated glomerular filtration rate [eGFR] <30 or requiring dialysis, or renal transplant), and use of osteoanabolic or antiresorptive medications for osteoporosis treatment were excluded.

### Study procedures and laboratory methods

Height and weight were measured by standard methods as published previously,^(^ ^18^, ^19^ ^)^ and body mass index (BMI) was calculated using height and weight data. History of diabetes and its treatment and diabetes complications were self‐reported. Up to 10 years of HbA1c was collected from electronic medical records.

A fasting morning blood sample was collected for measurement of serum calcium, phosphorus, creatinine, HbA1c (by HPLC, normal level <5.7%, precision <3%), 25‐hydroxyvitamin D (25‐OHD by Immunodiagnostic Systems [IDS; Boldon, UK] normal range 30–100 ng/mL, sensitivity of the assay 6 ng/mL, precision <9%), parathyroid hormone (PTH by IDS; normal range 11.5–78.4 pg/mL, sensitivity 5 pg/mL, precision <10%), and bone turnover markers (intact P1NP by IDS, normal range 27.7–127.6 ng/mL, sensitivity 2 ng/mL, precision <6%; CTX by IDS, normal range for postmenopausal females 0.142–1.351 ng/mL, sensitivity 0.08 ng/mL, precision <9%; and total osteocalcin by IDS, normal range 10.4–45.6 ng/mL for adults, sensitivity 2 ng/mL, precision <6%). All laboratory analyses were carried out at the Clinical Translational Research Center (CTRC) Core Lab at University of Colorado and details of assays and precision can be found at https://cctsiapps.ucdenver.edu/resources/services/pricelist/corelab.cfm?sc=15.

All participants underwent DXA for body composition and BMD at lumbar spine, total hip, and distal radius. Hologic (Marlborough, MA, USA) Discover W model was used during the initial part of the study and Hologic Horizon A in the later part of the study.

### Bone biopsy

Each subject was given in vivo double tetracycline labeling (500 mg three times per day orally on days 1–3 and days 15–17) as previously published.^(^ ^13^ ^)^ The biopsy was performed 5 days (±2 days) after the last label was administered. A transiliac biopsy was performed at a standard site approximately 2 cm posterior and inferior to the anterior superior spine using a trephine of 7.5 mm inner diameter (Medical Innovations International, Inc., Rochester, MN, USA). Bone samples were processed, embedded, sectioned, stained, and examined as previously reported.^(^ ^20^, ^21^, ^22^ ^)^

### Bone histomorphometry

Bone histomorphometry was performed by SQ at the Bone and Mineral Research Laboratory, Henry Ford Health, who was blinded to participants' diabetes status. Bone histomorphometric variables were designated in accordance with the nomenclature recommended by the American Society for Bone and Mineral Research^(^ ^23^ ^)^ and as previously reported by our group.^(^ ^24^, ^25^ ^)^

### Statistical analysis

Sample size estimation was based on feasibility and no power calculation was made for bone turnover analysis from histomorphometry data. Continuous data are presented as mean and standard deviations or median based on normality of distribution. Categorical data are presented as number and percentage. Baseline characteristics and histomorphometry variables were tested between women with and without diabetes by a Student's *t* test. Linear regression modeling was used to adjust histomorphometry variables by age and menopausal duration. Percentage differences between histomorphometric variables were calculated as T1D‐controls/(T1D + Controls/2) × 100. *p* < 0.05 was considered as statistically significant.

## Results

Of the 19 participants who underwent bone biopsy, samples could not be obtained from 3 participants (1 with T1D and 2 controls). Nine postmenopausal women with T1D and 7 controls without diabetes were included in the analysis. Baseline characteristics of the participants by diabetes status are shown in Table 1. Postmenopausal women with T1D had long‐standing diabetes of 38 years with mean 10 years of HbA1c of 7.5%. Seventy‐five percent of postmenopausal women with T1D reported at least one fracture compared with 43% of controls without diabetes.

**Table 1:** Baseline Characteristics of the Cohort

Variables	Postmenopausal women with T1D (*n* = 9)	Postmenopausal women without diabetes (*n* = 7)
Age (years)	58.4 ± 7.1	60.9 ± 3.3
Diabetes duration (years)	37.9 ± 10.9	NA
BMI (kg/m^2^)	28.9 ± 5.7	27.9 ± 6.9
Total lean mass (Kg)	43.2 ± 6.4	39.5 ± 4.8
Total hip BMD (gm/cm^2^)	0.863 ± 0.06	0.876 ± 0.07
LS BMD (gm/cm^2^)	1.01 ± 0.10	1.091 ± 0.144
Distal radius BMD (gm/cm^2^)	0.651 ± 0.07	0.713 ± 0.07
Menopause duration (years)	11.6 ± 9.8	8.0 ± 5.5
HRT use, *n* (%)	5 (55%)	4 (57%)
10‐year mean A1c	7.5 ± 0.7	
A1c at the time of screening	7.1 ± 0.4	5.4 ± 0.2*
Serum calcium (mg/dL)	9.4 ± 0.4	9.8 ± 0.2*
Serum phosphorus (mg/dL)	3.7 ± 0.6	3.9 ± 0.5
Urine calcium (mg/dL)	7.4 ± 4.1	8.5 ± 5.4
eGFR (mL/min/1.73 m^2^)^a^	74.6 ± 18.4	73.9 ± 9.0
Self‐reported any fractures	6 (75%)	3 (42.9%)
Presence of diabetic retinopathy, *n* (%)	7 (78%)	NA

*Note*: Data presented as mean ± SD or *n* (%).

Abbreviations: BMD = bone density by DXA; BMI = body mass index; eGFR = estimated glomerular filtration rate; HRT = hormone replacement therapy; LS = lumbar spine (L_1_ to L_4_); NA = not applicable; T1D = type 1 diabetes.

^a^ Two participants with T1D had eGFR between 30 and 60 mL/min/1.73 m^2^.

* *p* < 0.05.

Serum bone formation (P1NP, total osteocalcin), bone resorption (CTX) markers, and parathyroid hormone (PTH) were not significantly different between postmenopausal women with T1D and controls without diabetes. Serum 25 (OH) D levels were higher among women with T1D than controls without diabetes (Table 2).

**Table 2:** Bone Turnover Markers, Vitamin D, and PTH Levels Between Postmenopausal Women With T1D and Controls Without Diabetes

Variables	Postmenopausal women with T1D (*n* = 9)	Postmenopausal women without diabetes (*n* = 7)
Serum PTH (pg/mL)	38.6 ± 8.1	51.9 ± 23.9
Serum 25 (OH) D (ng/mL)	53.1 ± 20.8	30.9 ± 8.2*
Serum CTX (ng/mL)	0.4 ± 0.2	0.51 ± 0.34
Serum P1NP (ng/mL)	64.5 ± 26.2	87.3 ± 45.3
Serum total OC (ng/mL)	17.2 ± 9.1	26.2 ± 14.9

*Note*: Data presented as mean ± SD.

Abbreviations: CTX = cross‐linked C‐telopeptide of type 1 collagen; OC = osteocalcin; P1NP = procollagen type I N‐propeptide; PTH = parathyroid hormone; T1D = type 1 diabetes.

* *p* < 0.05.

Compared with postmenopausal women without diabetes, there were no differences in bone structural parameters such as bone volume as a percent of total volume (BV/TV), wall thickness(W.Th), osteoid thickness (O.Th), and trabecular measures of thickness (Tb.Th), number (Tb.N), or separation (Tb.Sp) in postmenopausal women with T1D (Table 3).

**Table 3:** Differences in Bone Structure Between Postmenopausal Women With T1D and Controls Without Diabetes

Variables	Postmenopausal women with T1D (*n* = 9)	Postmenopausal women without diabetes (*n* = 7)	Percent difference (T1D versus controls)	*p* Value
BV/TV (%)	17.2 ± 4.6	15.5 ± 2.6	+10%	0.4
W.Th (μm)	29.13 ± 2.06	29.37 ± 1.60	0%	0.8
O.Th (μm)	9.03 ± 2.71	8.46 ± 2.28	+7%	0.7
Tb.Th (μm)	111.4 ± 25.0	103.7 ± 4.9	+7%	0.4
Tb.N (mm)	1.56 ± 0.37	1.49 ± 0.22	+5%	0.7
Tb.Sp (μm)	558.40 ± 142.24	579.21 ± 103.10	−4%	0.7
Ct.Th (μm)	1.60 ± 0.64	1.60 ± 0.39	0%	0.99

*Note*: Data are presented as mean ± SD. No significant differences between the two groups were observed.

Abbreviations: BV/TV = bone volume per tissue volume; Ct.Th = cortical thickness; O.Th = osteoid thickness; T1D = type 1 diabetes; Tb.N = trabecular number; Tb.Sp = trabecular separation; Tb.Th = trabecular thickness; W.Th = wall thickness.

There were no statistically significant differences in the bone formation parameters such as osteoid volume (Ov/BV), osteoid surface (OS/BS), osteoblast surface (Ob.S/BS), and bone formation rate (BFR/BS or BFR/BV) among postmenopausal women with T1D compared with controls (Table 4). Activation frequency (Ac.F) was nonsignificantly lower among postmenopausal women with T1D, mainly attributable to reduced bone formation rate. The bone resorption parameters were not significantly different between the two groups (Table 4). Differences in bone structure and bone turnover parameters remained statistically nonsignificant even after adjustment for age and menopausal duration (data not shown). There were two participants with T1D who had eGFR between 30 and 60 mL/min/1.73m^2^. In a sensitivity analysis after removing these two participants, there was no difference in bone formation or resorption parameters (data not shown).

**Table 4:** Differences in Bone Turnover Between Postmenopausal Women With T1D and Controls Without Diabetes

Variables	Postmenopausal women with T1D (*n* = 9)	Postmenopausal women without diabetes (*n* = 7)	Percent difference (T1D versus controls)	*p* Value
Bone formation				
OV/BV (%)	1.00 ± 0.6	1.5 ± 1.3	−40%	0.3
OS/BS (%)	5.93 ± 3.54	8.44 ± 6.22	−34%	0.3
O.Th. (μm)	9.03 ± 2.71	8.46 ± 2.28	+7%	0.7
Ob.S./BS	1.31 ± 0.60	1.94 ± 2.34	−39%	0.4
Bone mineralization				
MS/BS (%)	2.71 ± 1.24	4.67 ± 3.56	−53%	0.2
MS/OS (%)	54.75 ± 32.20	55.35 ± 24.72	−1%	0.96
BFR/BS (%/yr)	6.13 ± 2.95	9.66 ± 7.40	−45%	0.2
BFR/BV (%/yr)	8.63 ± 3.97	14.43 ± 10.61	−50%	0.2
MAR (μm/d)	0.61 ± 0.08	0.55 ± 0.06	+10%	0.2
Mlt (days)	36.27 ± 20.13	33.30 ± 17.13	+8%	0.8
Bone turnover and resorption				
Ac.f (cycles/yr)	0.20 ± 0.08	0.32 ± 0.25	−46%	0.2
ES/BS (%)	1.01 ± 0.49	0.97 ± 0.58	+4%	0.9
Oc.S./BS (%)	0.29 ± 0.16	0.22 ± 0.14	+27%	0.3

*Note*: Data are presented as mean ± SD. No significant differences between the two groups were observed.

Abbreviations: Ac.f = activation frequency; BFR = bone formation rate; ES/BS = eroding surface; MAR = mineral apposition rate; Mlt = mineralization lag time; MS/BS = mineralizing surface; MS/OS = mineralizing osteoid; O.Th = osteoid thickness; Ob.s/BS = osteoblast surface; OC.s/BS = osteoclast surface; OS/BS = osteoid surface; OV/BV = osteoid volume; T1D = type 1 diabetes.

## Discussion

To the best of our knowledge, this is the first study evaluating bone histomorphometry in postmenopausal women with long‐standing T1D. The findings of our study demonstrate no effect of diabetes on bone structure, despite long duration of diabetes. T1D was associated with reduction in bone formation rate, albeit statistically nonsignificant, without much effect on bone resorption rate.

The nearly 40% reduction in bone formation rate observed in our study was similar to that hypothesized by Krakauer and colleagues.^(^ ^13^ ^)^ The study by Armas and colleagues^(^ ^14^ ^)^ reported no differences in bone structure and bone turnover similar to our study. Many but not all studies have shown reduced bone formation markers in people with T1D,^(^ ^5^, ^26^ ^)^ consistent with the low bone formation, although statistically nonsignificant, observed in our study. Chronic hyperglycemia and accumulation of advanced glycation end products have been implicated as potential reasons for low bone formation in people with diabetes.^(^ ^27^, ^28^ ^)^

Most but not all studies using HR‐pQCT have reported microstructural deterioration of trabecular bone and reduced cortical thickness at the distal radius among people with T1D^(^ ^29^, ^30^, ^31^, ^32^ ^)^ and such changes are more pronounced in those who have microvascular disease.^(^ ^29^, ^31^ ^)^ However, our study and a previous study^(^ ^14^ ^)^ using gold standard histomorphometry did not demonstrate any differences in bone structure. Most of our patients (78%) had diabetic retinopathy, a microvascular complication of long‐standing type 1 diabetes. None of our participants had severe renal insufficiency including proteinuria, and only one participant had clinical diagnosis of peripheral neuropathy. Previous studies have reported deterioration in microarchitecture in people with diabetic kidney disease and peripheral neuropathy.^(^ ^29^, ^31^ ^)^

We speculate that diabetes may have detrimental effects on bone material properties without affecting the bone structure. Despite 75% of our T1D cohort reporting a fracture, there was no difference in bone structure between T1D and control. We speculate that bone intrinsic material properties such as stiffness, hardness, and elasticity may be implicated in bone fragility. A previous study has reported trends toward stiffer and harder cortical and trabecular bone in people with T1D compared with controls.^(^ ^33^ ^)^ Studies in both T1D and T2D have reported increased advanced glycation end product (AGE) accumulation in bone leading to deleterious tissue changes.^(^ ^28^ ^)^ Further analysis of biopsies is needed to elucidate the role of material properties in increased fracture with T1D.

Our study has several limitations. We excluded patients with diagnosis of osteoporosis and taking antiresorptive or anabolic therapy, and thus, all selected patients were relatively healthy and free from metabolic bone disease. Vitamin D levels were higher in people with T1D than controls in our cohort, which may have reduced the magnitude of differences in bone formation and resorption between two groups. We did not analyze IGF‐1 and other hormones that may help us to explain some of the differences in bone structure and turnover between the two groups. Our T1D cohort was relatively well controlled with mean 10‐year HbA1c of 7.5%. It is possible that patients with suboptimal glycemic control would have significant deterioration in both structure and bone turnover. Our study was not powered to detect differences in bone histomorphometric parameters and the small sample size may have led to nonsignificant results. Bone biopsy is an invasive procedure, and thus, small sample size is an inherent limitation of most published studies on bone histomorphometry. We think that creating a bone biopsy sample repository and data sharing by various investigators working in this field may help to increase sample size and power to detect differences in bone structure and bone turnover.

In conclusion, long‐standing T1D in postmenopausal women may have an adverse effect on bone formation. Further research is needed to understand the effects of T1D on bone structure, turnover, and tissue material properties to elucidate mechanisms of bone fragility in this high‐fracture‐risk population with relatively near normal BMD.

## Author Contributions

**Viral N Shah:** Conceptualization; data curation; funding acquisition; investigation; methodology; project administration; resources; supervision; writing – original draft; writing – review and editing. **Shijing Qui:** Formal analysis; investigation; methodology; software; writing – review and editing. **Jason Stoneback:** Investigation; methodology; writing – review and editing. **Lubna Qamar:** Data curation; project administration; writing – review and editing. **Virginia L Ferguson:** Investigation; methodology; resources; writing – review and editing. **Wendy M Kohrt:** Conceptualization; investigation; methodology; resources; supervision; writing – review and editing. **Janet K Snell‐Bergeon:** Conceptualization; data curation; formal analysis; investigation; methodology; supervision; writing – review and editing. **Sudhaker D Rao:** Conceptualization; investigation; methodology; resources; supervision; writing – review and editing.

## Disclosures

The authors declare that they have no known competing financial interests or personal relationships that could have appeared to influence the work reported in this article.

### Peer Review

The peer review history for this article is available at https://www.webofscience.com/api/gateway/wos/peer‐review/10.1002/jbm4.10831.

## Data Availability

Investigators interested in collaboration using these study samples or data can e‐mail VNS at viral.shah@cuanschutz.edu. De‐identified data will be available after data user agreement with University of Colorado.
